# Societal allostatic load under chronic threat: defensive dominance and collective efficacy (Iran as a case example)

**DOI:** 10.3389/fnbeh.2026.1787124

**Published:** 2026-02-12

**Authors:** Jamshid Faraji

**Affiliations:** Canadian Centre for Behavioral Neuroscience, Department of Neuroscience, University of Lethbridge, Lethbridge, AB, Canada

**Keywords:** allostatic load, collective efficacy, human behavior, Iran, mental health, national allostatic load, stress, systems neuroscience

## Introduction

Chronic exposure to threat cues such as economic unpredictability, environmental hazards, institutional coercion, and persistent uncertainty can recalibrate human behavior at scale by biasing cognition and social interaction toward short-horizon, defensive strategies (Ruggeri et al., 2022; Batruch et al., 2025; Polasky et al., 2025). Within behavioral science, these adaptations are well described at the individual level (Valadez et al., 2022; Ying et al., 2023). The unresolved challenge, however, is to specify how such threat-induced adaptations aggregate across networks and institutions, potentially reshaping collective efficacy or the shared capacity to coordinate, cooperate, and sustain future-oriented public goods (Agneman et al., 2022; Lorenz-Spreen et al., 2023).

Current conditions in Iran offer a salient case for illustrating these dynamics, where population-level mental-health indicators and regional disparities may provide early, measurable signatures of escalating societal allostatic load. Although Iran serves as a concrete case, the proposed behavioral dynamics are expected to generalize to other settings marked by chronic threat exposure, including climate-stressed regions, conflict-affected societies, and weakly governed economies. Notably, the societal allostatic load framework discussed here is intended as a portable, testable hypothesis, but its expression should not be assumed to be uniform across countries. Political structure (e.g., competitive democracy vs. consolidated authoritarianism), institutional capacity (rule-of-law constraints, administrative competence, fiscal buffering), and information ecology (media freedom/censorship and digital fragmentation) are likely to moderate the magnitude, visibility, and dominant behavioral signatures of elevated societal allostatic load. Accordingly, Iran is used here to illustrate the proposed mechanisms; therefore, generalizability should be evaluated empirically in comparative designs that test whether inferred load predicts outcomes across settings and whether these moderators systematically shift lag structure and recovery trajectories.

Iran currently faces overlapping stressors spanning mental health, governance, ecology, and external security. Suicide rates are rising rapidly in Iran (Nouhi Siahroudi et al., 2025), and the regional distribution of suicide further shows that poorer, more rural provinces, often characterized by high out-migration and chronic underinvestment, now exhibit concentrated clusters of high-risk populations (Abbasi-Ghahramanloo et al., 2024). Other national indicators point to further challenges: The United Nations (UN) recently warned that Iran's execution rates have surged to a level that violates international human rights law (Commissioner, 2025). Ecological pressures mirror these social strains. Water bankruptcy is driving an unprecedented socioecological unraveling in Iran, one so severe that President Masoud Pezeshkian publicly acknowledged the crisis in late July 2025 (Rasheed, 2025). He warned that without drastic reductions in consumption, Tehran's reservoirs could be completely dry by September or October of the same year. Yet this declaration, striking as it is, represents only one facet of a broader systemic breakdown. On 13 June 2025, an Israeli airstrike breached Iranian airspace defenses and directly struck nuclear, missile-defense, and missile-production sites, an assault that damaged Iran's military infrastructure and its symbolic authority (Reuters, 2025). The World Bank, meanwhile, reports that Iran is facing recession, soaring inflation, and rising unemployment, with sanctions and internal mismanagement pushing many households into deeper economic distress (Bank, 2025). The Islamic Republic now confronts a cascade of interconnected environmental, economic, political, and institutional crises that reinforce one another and erode the state's residual adaptive capacity. Water scarcity, collapsing aquifers, massive and widespread land subsidence, persistent air pollution, which alone causes approximately 59,000 deaths per year (Motamedi, 2025), dust storm proliferation, and mass internal displacement are no longer discrete challenges; they are symptoms of a governance system overwhelmed by chronic stress, incapable of strategic coordination, and increasingly reliant on defensive reflexes rather than long-term planning. Indeed, these pressures form a coupled stressor environment consistent with the multi-driver conditions under which societal allostatic load would be expected to emerge. It is noteworthy that the news/NGO sources in the present opinion are used only to document event timing and contextual stressors; mechanistic claims are grounded in peer-reviewed theory and tested via the manuscript's model-based predictions.

A central consequence of chronic allostatic load (McEwen and Stellar, 1993) in neural systems is the progressive dominance of defensive survival circuits over integrative, outcome-based regulation. Under escalating stress, neural networks shift from flexible, goal-directed behavior toward rigid, self-preserving patterns that prioritize immediate threat avoidance, often regardless of long-term costs. This principle is strikingly reflected in Iran's current political trajectory, where the governing apparatus increasingly behaves less like a system tasked with managing a national organism and more like a defensive circuit protecting its own continuity.

Whereas functional political systems treat the public as the foundational *self* , legitimate dissent as informational feedback, and institutions as regulatory scaffolds, systems under heavy stress begin to reinterpret incoming signals through the prism of survival. In stress neurobiology, this resembles threat generalization, where circuits expand the category of *danger* to include previously neutral stimuli (McEwen and Morrison, 2013). Politically, this translates into perceiving civic actors as destabilizing forces rather than constituents. Indeed, political self-preservation in this context manifests as an inward collapse of the definition of the *self* that is worth protecting. Instead of safeguarding the population, the political system shifts to safeguarding a shrinking inner circuitry.

The analytic lens can be widened toward a clearer horizon. Recent political repression in Iran (intensified surveillance of dissidents, accelerated arrests and executions, expanded internet filtering, and heightened restrictions on public assembly) (Amnesty International, 2024; Hafezi and Rasheed, 2025; Brennan, 2026; Wallace, 2026) point to a governing apparatus operating under conditions of profound and escalating stress. Rather than exhibiting behaviors characteristic of a system engaged in adaptive governance, the Iranian state increasingly displays the hallmarks of a regulatory network dominated by defensive reflexes. Systems neuroscience offers a uniquely powerful framework for interpreting these patterns. When neural circuits face chronic and overwhelming stressors, they accumulate allostatic load, a concept describing the cumulative burden imposed on a system forced into sustained, energy-intensive states of vigilance. Under high allostatic load, neural systems undergo structural and functional deterioration that degrade regulatory control and bias behavior toward hyper-reactive, survival-oriented modes.

Here, drawing on my opinion as an Iranian behavioral neuroscientist, I argue that the Iranian political system exhibits analogous patterns of executive dysregulation, with consequences not only for governance but also for social well-being, scientific progress, and long-term systemic resilience. This level of dysregulation reflects a form of political self-preservation in which defensive circuits dominate the broader system. Beyond its societal manifestations, I will also discuss how this dysregulation impacts Iranian academia, science, and the broader scientific community. Indeed, several alternative accounts could generate similar surface patterns. Economic contraction, sanctions, demographic shifts, and changes in reporting, access to services, or censorship can each inflate or distort indicators such as help-seeking, suicide recording, protest visibility, and online sentiment. The present framework, therefore, should be read as a model-based hypothesis; its added value is not any single outcome, but the prediction of a coherent, cross-domain signature (co-movement across decision horizons, trust/cooperation, participation, and network connectivity) plus specific lag structures and non-linearities.

A key empirical test is whether a latent national allostatic load (NAL) model improves out-of-sample prediction relative to single-driver explanations (e.g., hardship-only), and whether inferred NAL(t) tracks behavioral shifts across independent data streams. The NAL is defined here as a latent, time-varying population-level state reflecting cumulative strain on a country's core regulatory institutions and social feedbacks under chronic threat distinct from individual allostatic load. The construct is theoretically useful because it captures cross-domain coupling and persistence that single-driver stress models (e.g., economic hardship only or repression only) often treat as separate or transient. In this framework, elevated NAL is hypothesized to undermine collective efficacy (a population's capacity for shared problem-solving and coordinated action) through three intertwined pathways: (*i*) trust erosion (reduced willingness to cooperate and accept uncertainty), (*ii*) coordination frictions (weakened institutional bandwidth, degraded information flow, and higher transaction costs), and (*iii*) shortened temporal horizons (greater preference for immediate safety/utility over long-run public goods). These shifts reduce the feasibility of cooperative equilibria, amplifying defensive, fragmented behavioral regimes.

Furthermore, in the present Opinion, the evidence types are kept distinct. Peer-reviewed behavioral and political science motivates the proposed mechanisms and predicted signatures, whereas news and NGO reporting is used primarily to document event timing and salient contextual shifts. The central claim is, therefore, evaluated by whether the predicted cross-domain signature and lag structure emerges in independent datasets, rather than by any single report or indicator. Notably, the correspondences developed here are intended as conceptual, functional analogies rather than literal “scaling up” of neural mechanisms to societies. The aim is to capture dynamical similarities (e.g., feedback breakdown, nonlinearity, hysteresis, stability-flexibility trade-offs) that can emerge in multi-level regulatory systems under chronic threat. Accordingly, the framework stands or falls on observable population/institutional signatures and model comparison, not claims of neural isomorphism.

## Chronic stress and breakdown of regulatory control

Neuroscience has long established that chronic stress reshapes the architecture and function of the brain's regulatory hubs. Prolonged activation of threat circuits induces prefrontal-limbic decoupling, a process in which top-down prefrontal control weakens while limbic structures (especially the amygdala) become hyperactive (McEwen, 2012; McEwen and Morrison, 2013; Liu et al., 2020). This decoupling leads to diminished executive function, impaired decision-making, reduced behavioral flexibility, and exaggerated threat perception. The stressed brain becomes less capable of contextualizing stimuli or distinguishing benign from harmful signals. Indeed, these mappings are intended as heuristic, testable correspondences, not literal neural equivalences at the population level. A similar pattern is currently visible in Iran's contemporary political behavior. I cite these events only to characterize the chronic-threat environment that the NAL framework seeks to model. Rather than engaging in deliberative governance (e.g., consulting experts, cultivating institutional autonomy, investing in long-term planning), the system increasingly relies on reflexive defensive activation (Tajali, 2023; Barjasteh, 2025; Iran, 2025; Tohidi and Daneshpour, 2025; Watch, 2025a). Groups integral to civic vitality (i.e., academics, reformists, women's rights advocates, ethnic communities, journalists, and even non-politicized segments of youth culture) are reclassified as latent threats. This threat generalization mirrors stress-induced reductions in neural discriminative precision. The political analog to prefrontal-limbic decoupling is the eclipse of deliberative institutions (parliament, ministries, courts) by coercive organs (intelligence services, security forces). As coercive circuits dominate, the system becomes trapped in metabolically costly, cognitively rigid survival strategies. Much like stressed neural networks overreact to benign cues, Iran's political system increasingly interprets routine civic activity such as assemblies, discourse, and scientific collaboration as existential dangers requiring suppression.

## Political self-preservation as defensive circuit dominance

A hallmark of chronic allostatic load in neural systems is the dominance of survival circuits over integrative ones (McEwen and Morrison, 2013; Zeng et al., 2024). Stressed neural networks abandon flexible, goal-directed behavior for rigid self-preservation. Iran's political system reflects this shift with striking fidelity. Healthy neural and political systems define the *self* broadly, encompassing diverse circuits or, politically, the population and its institutions. Under chronic stress, however, systems contract the definition of the self, protecting only the central machinery sustaining the defensive reflex. In present-day Iran, this manifests as prioritizing security organs (e.g., the IRGC and Quds Force), gatekeeping ideological institutions (e.g., the Guardian Council), and patronage networks (e.g., the Basij and bonyads). This narrowing closely parallels threat generalization in stress neurobiology (McEwen and Morrison, 2013). Political self-preservation then reorganizes governance architecture itself. Integrative institutions (universities, technocratic ministries, regulatory agencies) are subordinated to defensive imperatives. Their decision-making capacity atrophies, resembling dendritic retraction in chronically stressed prefrontal neurons.

As defensive circuits dominate, resource allocation shifts away from education, innovation, public health, and scientific development toward surveillance and coercive capacities. This produces a low-plasticity political environment marked by rigidity, low tolerance for complexity, and truncated time horizons. The ultimate outcome is autopoietic defensive recursion: repression generates instability, which demands more repression. This self-reinforcing loop echoes pathological positive-feedback phenomena in neural and immune systems. Political self-preservation thus becomes a source of accelerated fragility and increases the probability of systemic destabilization (Aytaç et al., 2017; Olsson et al., 2018; Hellmeier et al., 2019; Earl et al., 2022).

## Network fragmentation and loss of integrative capacity

Chronic stress degrades white-matter tracts and long-range connectivity, impairing integrative communication across neural networks (Goldwaser et al., 2021; Hardi et al., 2025). Political systems exhibit analogous fragmentation when judicial, academic, and technocratic channels erode. In Iran, decision-making has become increasingly siloed, with fragmented and overlapping institutions competing for influence rather than contributing to coherent, integrated governance (Siavoshi, 2025). For instance, Dr. Ahmadreza Djalali, an Iranian-Swedish disaster-medicine scientist who was formally invited to Iran by the University of Tehran for academic collaboration, was arrested by IRGC intelligence agents in April 2016 and has remained imprisoned ever since, held under a standing death sentence despite repeated international appeals (Amnesty International, 2025). It appears that expertise-based institutions in Iran weaken; loyalty-based appointments proliferate; and state-society interfaces degrade. The system loses its political white matter, the connective infrastructure enabling coherent governance, coordinated planning, and error correction. Consequences include contradictory policy signals, incoherent responses to crises, degraded environmental and public-health planning, and weakened economic stabilization. Analogous patterns have been observed in other systems under chronic stress, such as Venezuela's post-2015 governance crisis (The, 2018; Roy and Cheatham, 2024), Syria's institutional erosion during the civil war (Alaref et al., 2023), and Sri Lanka's 2022 fiscal collapse (Niriella et al., 2025), each reflecting identifiable signatures of defensive dominance and network fragmentation.

## Analogy: disconnection syndromes

Modern immunology and neuroscience converge on the pathology of Multiple Sclerosis (MS), where autoreactive T cells infiltrate the central nervous system and strip myelin sheaths, degrading white-matter integrity and long-range communication (Figueroa-Vargas et al., 2025; Orr and Steinman, 2025). The resulting conduction block, slowed signaling, and network fragmentation diminish the brain's ability to integrate distributed information and coordinate adaptive behavioral responses. This pathophysiology parallels Iran's progressive erosion of institutional myelin: independent courts, universities, civil regulatory bodies, and technocratic ministries that once insulated and facilitated effective system-wide communication (Watch, 2025b). As autoreactive immune responses in MS undermine core integrative pathways, Iran's political system similarly disrupts its own connective infrastructure through distrust, purges, and politicization. The outcome in both systems is a marked reduction in integrative capacity, increased local noise, and a shift toward maladaptive isolated responses rather than coherent, coordinated regulation. Analogously, and without implying a literal clinical equivalence, the societal patterns described here can be read as a “disconnection” phenomenon, resembling how demyelination in MS disrupts integrative signaling, where chronic stressors degrade the coordination and information flow needed for coherent, future-oriented collective action.

## Weakened science, isolated scientists, and disconnected knowledge systems

Scientific ecosystems depend on connectivity, autonomy, and open intellectual exchange. Rising allostatic load in Iran weakens all three. Universities face surveillance, restricted travel, filtered digital access, and politicized funding. Many researchers experience interrogation, dismissal, or censorship. These conditions reduce scientific risk-taking, narrow research agendas, and weaken exploratory thought. Iran's scientific networks undergo sociopolitical synaptic pruning, weakening global connectivity needed for innovation. Long-term consequences include scientific brain drain, decreased output, and reduced international scientific engagement (Stone, 2018). Empirical indicators now confirm this functional decline in Iran. According to SCImago-based statistics cited by Iran's own science-policy officials, the country's global ranking in scientific publications has slipped from 15th to 17th over the past 3 years (Akhoundzadeh, 2025). Meanwhile, reporting in Science documents an accelerating collapse of international connectivity: increasing digital isolation, the expansion of national intranet controls, the criminalization of VPN use, and shrinking access to global journals and collaborations have fundamentally undermined Iran's integration into the global research ecosystem (Stone, 2023). This scientific degradation also mirrors the structural disconnection observed in degenerative brain conditions. In demyelinating disease, the loss of myelin disrupts not only the speed of neural transmission but also the synchrony required for distributed cognitive processing, leading to impaired learning, reduced adaptability, and weakened higher-order integration (Shimizu et al., 2023; Mercier et al., 2024; Murayama et al., 2025). Iran's scientific system is exhibiting a parallel decline in functional connectivity: barriers to data exchange, erosion of institutional autonomy, and the erosion of international partnerships (Stone, 2023) collectively compromise the nation's research white matter. As demyelinated neural networks lose the capacity for coordinated information flow, Iran's fragmented scientific infrastructure struggles to generate sustained innovation, evaluate complex national challenges, or contribute meaningfully to global knowledge production.

International sanctions function as an exogenous source of allostatic load. External sanctions impose chronic structural stress on the population, institutions, and governance system. Sanctions degrade health systems, increase mortality, disrupt medical supply chains, and heighten mental-health burdens (Pinna Pintor et al., 2023; Mohamadi et al., 2024). In Iran, sanctions restrict access to medicines and diagnostic technology, worsening outcomes in cancer, cardiovascular disease, and chronic illness (Gorji, 2014; Karimi and Haghpanah, 2015; Haghjou et al., 2024). Economic contraction and deteriorating living conditions are closely associated with heightened psychological distress and suicidality in Iran (Kakaei et al., 2023; Rouzrokh et al., 2025). For science, however, sanctions create a scientific disconnection syndrome (blocked banking and grant access, reagent and equipment shortages, reduced international mobility, constrained collaboration) (Stone, 2018; Naghavi-Shoae, 2025). This enforced isolation closely mirrors neural disconnection syndromes.

## Runaway defensive dynamics and allostatic destabilization

Neuroscience identifies runaway feedback loops (e.g., seizure-like cascades, hyperinflammatory storms, and other forms of uncontrolled network activation) as consequences of lost inhibitory control under chronic stress (Akil and Nestler, 2023; Nusslock et al., 2024). Iran's repression cycles exhibit a similar loss of systemic inhibition: each crackdown generates public unrest, which in turn provokes intensified coercion, locking the political apparatus into a self-reinforcing loop of defensive escalation. As judicial oversight, legislative accountability, and press autonomy erode, the system loses its inhibitory circuitry and becomes unable to downregulate activation even when repression becomes counterproductive. The recent Israeli attack further exposed this vulnerability. Rather than producing coordinated crisis governance, the shock induced fragmentation, perception management, and elite self-protection, behaviors characteristic of overstressed neural circuits that misclassify internal signals as external threats. In this state of elevated allostatic load, dissent is interpreted as danger, routine civic expression is pathologized, and defensive reflexes intensify irrespective of long-term systemic cost, signaling a drift toward allostatic collapse.

## Competing accounts and discriminating predictions

Indeed, several single-factor accounts could plausibly explain elements of the observed behavioral and institutional shifts, including economic contraction and inflation, sanctions and trade shocks, repression and information control, demographic change, and reporting bias. These accounts are valuable and may be sufficient for specific outcomes (e.g., hardship-driven migration, sanction-linked shortages, repression-linked protest suppression, or apparent changes due to measurement artifacts). However, I argue that they often struggle to explain cross-domain coherence (i.e., coordinated shifts across multiple behavioral streams with consistent lag structure) as well as persistence and non-linearity (hysteresis, threshold effects) after repeated shocks. The NAL framework, therefore, is offered here as an integrative alternative: it predicts that diverse stressors converge on a latent, time-varying state that couples behavioral, institutional, and social-feedback dynamics. Empirically, these models can be contrasted by asking whether (a) a single-driver covariate explains most variance across outcomes, or (b) a latent NAL(t) model better captures joint dynamics, lagged propagation, and recovery trajectories. Evidence favoring NAL would include stronger cross-stream co-movement than any single driver predicts, consistent lags across domains, and recovery hysteresis even when a focal stressor partially abates.

**Figure 1:**
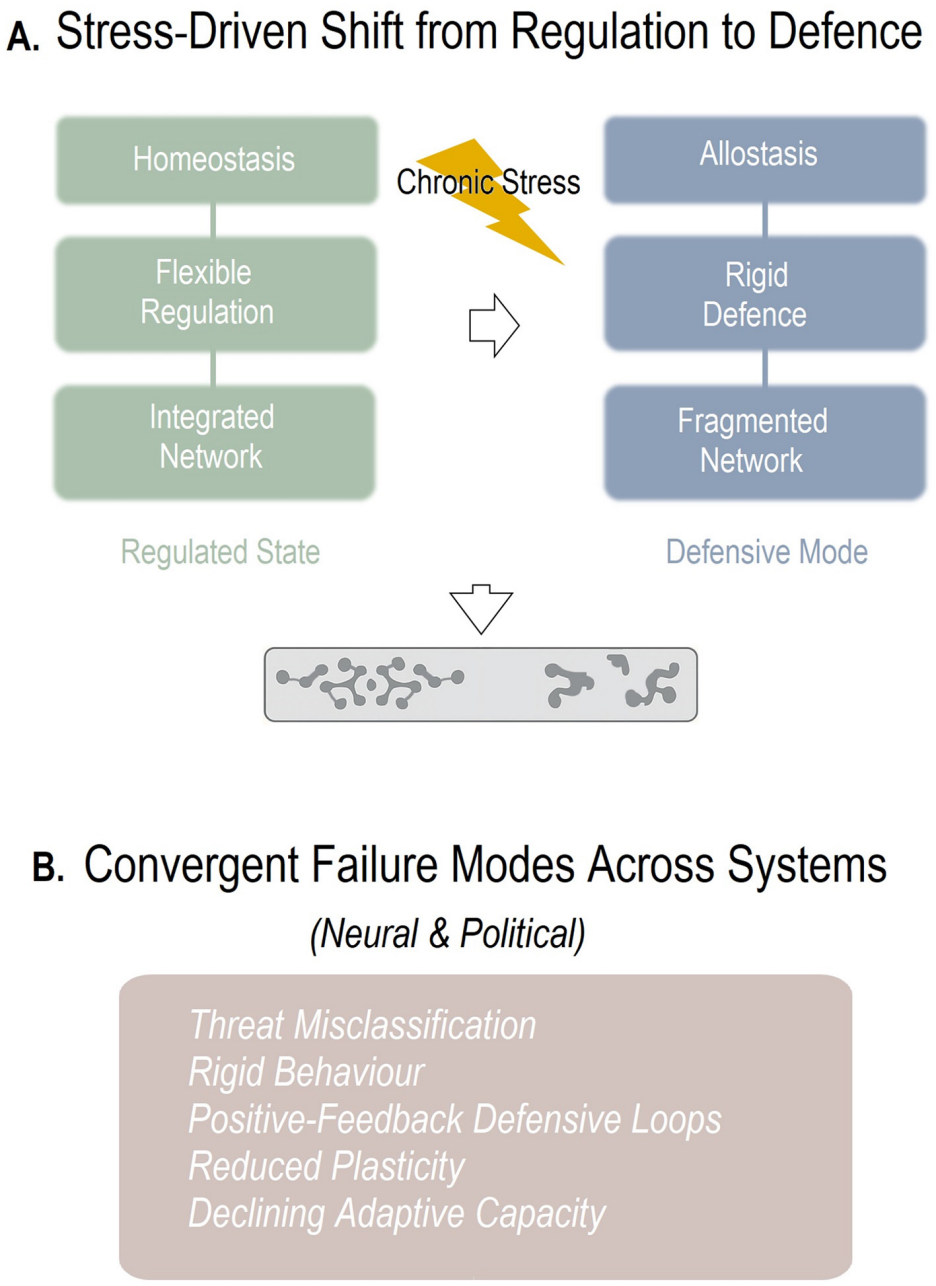
Heuristic model linking chronic threat, NAL, collective efficacy, and behavioral signatures. **(A)** Chronic stress pushes regulatory systems (whether neural or political) from flexible, integrative network states toward rigid, fragmented defensive modes. Left: regulated systems exhibit homeostasis, flexible top-down control, and coherent integrative connectivity. Right: chronic stress induces allostatic strain, defensive rigidity, and loss of network coherence, mirroring the transition observed in Iran's political system under legitimacy stress. **(B)** Despite their differences, neural circuits under prolonged allostatic load and political systems under chronic legitimacy stress exhibit shared patterns of failure. These include threat misclassification, behavioral rigidity, self-reinforcing defensive activation, reduced plasticity, and declining adaptive capacity, hallmark features of systems undergoing progressive dysregulation.

Taken together, a systems neuroscience framework reveals how chronic stress destabilizes Iran's political system. High allostatic load degrades regulatory control, fragments connectivity, and erodes scientific capacity; by analogy, this resembles prefrontal-limbic decoupling under chronic stress, while sanctions intensify the overall load. Systems, neural or political, cannot remain stable when defensive circuits prioritize a narrowing core over the broader network (Figure 1). Iran's political dynamics now exhibit hallmark features of advanced load-induced dysregulation: hyperreactive threat processing, disrupted integrative pathways, diminished plasticity, and runaway defensive escalation. Breaking this defensive spiral will require a shift toward political rehabilitation grounded in restored regulatory balance and reduced systemic stress. If systems biology offers any lesson, it is that stability in chronically stressed networks is restored only by reactivating regulatory pathways, rebuilding lost connectivity, and reducing the load on defensive circuits. Of note, the neural-societal mapping in the present opinion is a heuristic for generating falsifiable, pattern-level predictions, not a claim of literal neural mechanisms at the societal scale. Thus, the framework should be judged by comparative model performance rather than by the analogy's rhetorical appeal. For Iran, this implies strengthening deliberative institutions, reopening scientific and civic channels, and alleviating the systemic pressures that perpetuate defensive overactivation.

Practically, the NAL framework can guide design and evaluation of interventions by treating population instability as a coupled, cross-domain state rather than a single-sector problem. Policy levers map to three targets: (1) *load reduction* (buffer predictable stressors and uncertainty), (2) *feedback restoration* (strengthen deliberative, oversight, and service-delivery functions that downregulate defensive escalation), and (3) *connectivity rebuilding* (protect scientific/civic channels and prosocial coordination). More importantly, for public mental health, this implies prioritizing capacity where inferred NAL(t) is highest (e.g., regions showing convergent rises in help-seeking, withdrawal, volatility), and evaluating strategies by whether they reduce inferred NAL(t) and restore cross-stream coherence rather than merely suppressing visible unrest.

Moreover, although illustrated with Iran, the proposed NAL dynamics are expected to generalize to other settings characterized by sustained, overlapping stressors, and are best evaluated through comparative, cross-national tests of the discriminant signatures outlined here. A complementary model-based evaluation framework (Box 1) formalizes NAL as a latent population state that can be inferred from stressor streams and behavioral outputs, enabling explicit tests of nonlinearity, hysteresis, and recovery rather than relying on narrative correspondence alone. Ultimately, no system recovers through amplified defense; recovery emerges through the restoration of regulation, connectivity, and adaptive capacity.

A model-based evaluation framework for National Allostatic Load (NAL).

This box summarizes a proposed framework and specifies how future work can empirically test NAL using discriminant signatures. It formalizes national allostatic load (NAL) as a latent, time-varying population-level state that is not directly observed but can be inferred from convergent indicators across stressor exposure and behavioral/institutional outputs. In a minimal state-space formulation, exogenous stressors drive an unobserved NAL(t) which, in turn, shifts a small set of behavioral parameters (e.g., temporal discounting, ambiguity intolerance, threat generalization, and the perceived payoff of cooperation vs. withdrawal). These parameter shifts generate observable signatures (including reduced civic participation, increased help-seeking, polarized discourse, compliance oscillations, and protest–fatigue cycles) while enabling explicit tests of lag structure, dose-response, and heterogeneity across regions and strata.

### Minimal model (state-space sketch)

*- Inputs (drivers)*: economic volatility, ecological shocks, coercive events, information restriction (plus any other measurable threat indices).

*- Latent state*: NAL(t) where t denotes time, with inertia and shock sensitivity.

*- Outputs*: time series of behavioral indicators; optional mediators (trust/cooperation metrics where available).

### Observable outputs (examples)

Turnout/participation; mobility and social mixing; service use/help-seeking; sentiment and polarization; strike/protest incidence and decay (fatigue); compliance volatility; proxy measures of trust/prosociality.

### Falsifiable predictions

- *Non-line*arity/criticality: variance and network fragmentation increase disproportionately as inferred NAL(t) approaches a critical zone.

*(As inferred NAL(t) increases, behavioral disruption should rise disproportionately rather than linearly. Near a critical zone, small additional stressor changes should produce large jumps in outcomes such as volatility, polarization, or network fragmentation)*.

- Hysteresis: identical shocks produce larger effects and slower recovery under higher NAL(t) (recovery is not the reverse path).

*(System responses should be path-dependent, that is, the same shock should produce larger effects and slower recovery when baseline NAL(t) is higher than when it is low. When stressors abate, behavioral and institutional indicators should not immediately return to prior baselines, implying delayed and asymmetric recovery)*.

- Cross-stream coherence: independent behavioral streams co-move with NAL(t) after plausible lags.

*(Independent behavioral streams (e.g., participation, mobility, help-seeking, discourse) should co-move with inferred NAL(t) after plausible lags. A latent NAL(t) estimated from one subset of streams should predict changes in the others, supporting NAL as a common integrator rather than separate coincident trends)*.

- Model competition: if “single-driver” models (e.g., hardship-only) fit/predict as well without NAL(t), the NAL account is weakened; if not, NAL earns parsimony.

*(Models that include a latent NAL(t) should outperform single-driver alternatives (e.g., hardship-only) in out-of-sample prediction and parsimony-adjusted fit. If simpler models match performance without NAL(t), the NAL construct is weakened; if NAL adds predictive gain without overfitting, it earns explanatory value)*.

### Evaluating recovery

Interventions should be judged by whether they lower inferred NAL(t) and restore coupling between feedback, deliberation, and prosocial coordination, not merely suppress visible instability.
